# Severe Sepsis with Multiorgan Failure due to Melioidosis: A Lesson to Learn

**DOI:** 10.1155/2021/5563214

**Published:** 2021-04-08

**Authors:** Dinuka S Warapitiya, Shyama Subasinghe, Rukshanie Frances de Silva, Dadallage Lalitha Piyarisi, Kushlani Jayatilleke

**Affiliations:** ^1^Post Graduate Institute of Medicine, University of Colombo, Colombo, Sri Lanka; ^2^Sri Jayewardenepura General Hospital, Nugegoda, Colombo, Sri Lanka

## Abstract

**Introduction:**

Melioidosis is a bacterial infection caused by a Gram-negative bacillus *Burkholderia pseudomallei*, prevalent in Southeast Asia and Northern Australia. Sri Lanka is situated in the endemic belt of melioidosis. Melioidosis has a wide spectrum of clinical presentations and results in high mortality rates in severe infection. *Case Report*. We report a 54-year-old previously healthy Sri Lankan farmer who presented with septicemia following a cut injury to the right leg while working in a paddy field. Initially, he had mild wound sepsis, and later, his condition deteriorated rapidly. The patient required organ support later for cardiovascular instability, acute liver failure, acute kidney injury, acute respiratory distress syndrome, and coagulopathy. The patient's blood culture was negative on the admission day, and the repeated blood culture taken at the ICU was contaminated with a commensal flora initially and later isolated *Burkholderia pseudomallei*. Although wound swab culture taken on the first day isolated an organism, it took six days to identify it as *Burkholderia pseudomallei*. The patient succumbed to severe melioidosis leading to a severe sepsis and multiorgan failure in spite of treatment with meropenem.

**Conclusion:**

This case report highlights the importance of considering melioidosis as a differential diagnosis when a patient comes with risk factors for melioidosis.

## 1. Introduction

Melioidosis is an infection caused by *Burkholderia pseudomallei* which is a Gram-negative, oxidase-positive, aerobic, non-spore-forming motile bacillus that is found in soil and water. Melioidosis is common in the tropical and subtropical zone between 30°N and 30°S [1]. Melioidosis is considered as endemic in Sri Lanka.

There are three modes of transmission of this agent. They are inhalation, ingestion, and direct inoculation. Person to person, sexual, perinatal, vertical, and nosocomial transmission have also been reported [2, 3].

Clinical presentation of melioidosis infection ranges from an asymptomatic state to rapidly severe sepsis [2]. It also has a chronic and relapsing course and can remain dormant for long periods [2]. Polysaccharide capsule and lipopolysaccharide of the bacteria are resistant to compliment-mediated killing, which increases its virulence [2, 3].

Severe infection is common among people with comorbidities such as diabetes mellitus, renal disease, liver disease, alcohol consumption, and thalassemia. Occupational exposure to surface water and mud in the form of agriculture, farming, fishing, gardening, land surveying, building construction, and manual labouring are risk factors for melioidosis [4, 5]. A study conducted in Sri Lanka reported melioidosis in Sri Lanka has wide geographical distribution and it is common in rice-growing rural areas [1].

The incubation period of melioidosis varies, usually from 2 to 4 weeks, but can vary from 1 day to a few years. Clinical manifestations of melioidosis are not distinctive. Therefore, definitive diagnosis requires laboratory confirmation by isolation of *Burkholderia pseudomallei* from the clinical specimens. High degree of clinical suspicion is necessary to arrive at a diagnosis and commence treatment early because severe melioidosis is often lethal [1].

This case report highlights the importance of considering melioidosis in high-risk groups of Sri Lanka for early diagnosis and treatment to timely control the sepsis due to melioidosis.

## 2. Case Presentation

A previously healthy 54-year-old farmer from the Kurunagala district of Sri Lanka presented to our hospital with a history of fever with chills and rigors and a right leg wound following a cut injury and associated painful right leg swelling of 10 days duration. Twelve days back, he had sustained the cut injury from a cutting machine while he was walking in a paddy field. The wound was painful and had pus discharge. Along with fever, he had a painful inguinal lymph node on the right side. He had constitutional symptoms such as loss of appetite and malaise. Other systemic reviews were normal. He had consumed alcohol occasionally, and he was a nonsmoker.

Initially, he was treated by a general practitioner with oral antibiotics, and later, he was given in-ward treatment at a nearby local hospital for five days. At the local hospital, he was treated for febrile wound sepsis with intravenous cefotaxime 1 g 8 hourly and intravenous flucloxacillin 500 mg 6 hourly. Incision and drainage was performed for the wound at the local hospital. Despite the treatment, his symptoms worsened. Due to the lack of response, he got admitted to our hospital.

On the day of admission to our hospital, he was febrile. There was a superficial wound with pus on the anterior aspect of the right leg 5 cm proximal to the ankle joint which was associated with surrounding right-leg cellulitis. There was a tender right inguinal lymph node. Cardiovascular, respiratory, and abdominal examination findings were normal.

His investigations showed raised white cell count (WBC) (14,000 cells/mm^3^ 88% of neutrophils). The haemoglobin level was 12.4 g/dl, and platelet was 204,000/mm^3^. Blood picture showed neutrophil leukocytosis with left shift and toxic changes in neutrophils suggestive of a bacterial infection. C-Reactive Protein (CRP) was 173 mg/L. The Erythrocyte Sedimentation Rate (ESR) was 18 mm/hour. His liver function and serum creatinine were within normal limits. An ultrasound scan of the abdomen showed a large right inguinal lymph node without formation of an abscess. Blood culture and urine culture taken on the admission day were negative. A wound swab culture taken on the admission day isolated an organism; however, it took six days to identify the organism as *Burkholderia pseudomallei.*

Initially, he was treated with intravenous ceftriaxone 1 g twice a day. The wound was cleaned and dressed with povidone iodine.

Five days after in-ward treatment in our hospital, his general condition deteriorated. He complained of worsening dry cough and shortness of breath. There was intermittent fever at 99°F, and he complained of reduced urine output. He became tachypnoeic (respiratory rate 25 breaths/minute), dyspnoeic, and tachycardic (pulse rate 144 beats/minute). The systolic blood pressure was 116/60 mmHg. He had bibasal fine and coarse crepitations more on the left side. His oxygen saturation dropped to 93% on room air. There were no audible cardiac murmurs. Abdominal and neurological examination findings were normal. His condition progressively deteriorated out of control. He was taken into an intensive care unit (ICU) on the same day for further management. At the ICU, his condition further deteriorated. He developed type 1 respiratory failure and vasodilatory shock. He was promptly intubated and ventilated and started on inotropes.

On the day of admission to the ICU, all the blood investigations, cultures, and imaging studies were repeated. He had rising WBC (23,000 cell/mm^3)^ with neutrophil leukocytosis. Blood picture showed left shift and toxic changes in neutrophils without evidence of microangiopathic hemolytic anemia. There was a drastic drop in the platelets (112 × 10^3^/mm^3^). His CRP had risen to 420 mg/L, ESR was 20 mm/hour, and serum creatinine had increased to 356 micromoles/l with blood urea 81 mg/dl. He had oliguria. After admitting to the ICU, he was started on broad-spectrum antibiotics. Intravenous meropenem 1 g 12 hourly was started on day 1 of ICU suspecting possible melioidosis because of his exposure history with soil in the paddy field. A meropenem 1 g 12 hourly drug regime was given considering his acute kidney injury. He was also started on intravenous teicoplanin 400 mg daily to cover Gram-positive microbes and started intravenous metronidazole 500 mg three times a day to cover anaerobes and oral doxycycline 100 mg twice a day to cover atypical organisms.

During the ICU stay, after commencing on broad-spectrum antibiotics, his CRP came down to 350 mg/L, but his WBC remained around 15,000–20,000 cells/mm^3^. He had rising serum creatinine and blood urea and developed anuria. The serum lactate level increased up to 8 mmol/L. He had rising AST, ALT, and ALP levels. Prothrombin time, activated partial thromboplastin time, and International Normalized Ratio (INR) levels increased rapidly. His acute kidney injury was complicated with hyperkalemia. Creatinine phosphokinase was 94 U/L. The summary of investigation findings on day 01 and during the ICU stay is illustrated in Table 1.

**Figure 1:**
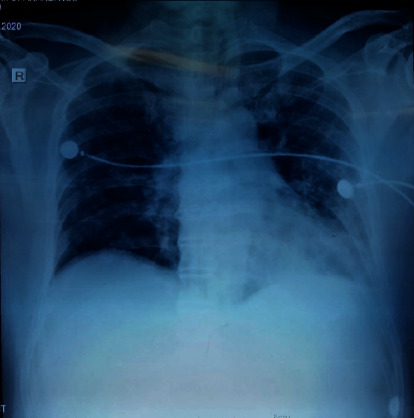
Chest X-ray showing heterogenous bilateral, bibasal opacification mainly on the left side.

Chest X-ray showed heterogenous opacifications at lung bases probably due to pulmonary oedema following acute kidney injury and due to associated melioidosis pneumonia (Figure 1). These X-rays features improved slightly while he was in the ICU. Transthoracic echo did not show vegetations. An ultrasound scan of the abdomen showed increased echogenicity of the kidney due to acute kidney injury.

Repeat blood culture, which was taken on day one of ICU stay, was initially contaminated with Gram-positive *Staphylococcus coagulase* negative organism which masked the *Burkholderia pseudomallei.* However, later, it isolated *Burkholderia pseudomallei*. On the 2^nd^ day of ICU stay, the positive blood and wound swab culture reports for *Burkholderia pseudomallei* were available. Then, the diagnosis of melioidosis with evidence of severe sepsis and multiorgan failure was made. Its sensitivity pattern showed resistance to ceftazidime, tigecycline, colistin, and piperacillin/tazobactam. However, it was sensitive to meropenem and trimethoprim/sulfamethoxazole. Intravenous meropenem was continued since it is the drug of choice for severe melioidosis.

Furthermore, he developed fast atrial fibrillation. He had progressively worsening acute kidney injury, acute liver failure, and septic shock while in the ICU. He had bleeding from the endotracheal tube and developed melaena due to coagulopathy and low platelet. The patient was on ventilation and with a support of inotropes till the end. He was treated with amiodarone when he developed fast atrial fibrillation. He underwent daily hemodialysis for acute kidney injury but hardly had any improvement. Blood products were given when he developed bleeding to correct coagulopathy and low platelets. However, surprisingly, his right leg wound and cellulitis resolved gradually. Despite all these interventions, he continued to deteriorate. He went into refractory septic shock and multiorgan failure and died on the 7th day of ICU stay. The cause of death was severe melioidosis septicemia with multiorgan failure.

Before positive culture reports were available, we had a few differential diagnoses apart from melioidosis when he was admitted to the ICU. Leptospirosis was one of the differential diagnoses. It is a zoonotic infection in Sri Lanka caused by a spirochetes of the genus Leptospira. Usually, the organism is acquired by direct inoculation on to a past skin abrasion or mucosa from the wet muddy soil such as from the soil in a paddy field. This is characterized by febrile illness, which is complicated with low platelet count, acute kidney injury, pulmonary haemorrhages, and sometimes, acute liver failure. However, our patient's leptospirosis IgM and IgG antibodies levels measured on the 2^nd^ week of the illness were negative. We also thought of osteomyelitis of the right tibia with associated wound sepsis due to methicillin-resistant *Staphylococcus aureus* (MRSA). However, blood culture did not show MRSA and X-rays of the right tibia and fibula were normal. Since he had signs of lung infection, we investigated him for COVID-19, but the COVID-19 PCR test was negative. His dengue antibodies were negative. Dengue is an endemic mosquito-born viral disease in Sri Lanka. Venous duplex of the lower limb was performed to exclude deep vein thrombosis, and it only showed right leg subcutaneous oedema. HIV 1 and 2 antibodies were negative.

This case was an example for severe melioidosis sepsis which led to rapid progression leading to multiorgan failure and death.

## 3. Discussion

Melioidosis is caused by Gram-negative saprophyte, *Burkholderia pseudomallei*, which is a very virulent organism and shows antibiotic resistance. Melioidosis can affect any organ of the body [6]. Its presentation ranges from acute to chronic and local to systemic or subclinical infection. If present as septicemia, it can progress to multiorgan failure and death. The mortality rate is high, 90% without effective treatment and 50% even after antibiotic therapy [7]. The study conducted in Sri Lanka using 250 culture-positive melioidosis cases reported from 2006 to 2017 May has shown the mortality was 20.4% in the study [8].

Severe infection is common in patients with diabetes, renal disease, liver disease, alcohol consumption, and thalassemia [5]. People who have exposure to wet soils or water such as farmers, manual labourers, workers of building construction sites, and surveyors or who are involved in gardening and fishing are at a high risk of getting *Burkholderia pseudomallei* infection [5]. This patient, although he was immunocompetent, had occupational exposure. The patient was a previously healthy farmer and recently had a cut injury from a cutting machine while working in a paddy field. Direct inoculation of the organism on the wound via damaged skin from the wet soil and water in the paddy field might be the mode of transmission of the infection. High degree of suspicion of melioidosis is needed when the patient is from an endemic area with a potential exposure history [5, 9, 10].

Melioidosis commonly affects the lungs followed by the skin and subcutaneous tissues [6, 11, 12]. Skin and soft tissue infection due to melioidosis accounts for 13%–24% of clinical presentations [6]. A study conducted in Sri Lanka has shown that lung infection due to melioidosis predominated followed by musculoskeletal infections, abdominal involvement, and skin and soft tissue involvement [8]. Local infection of the skin and subcutaneous tissue due to melioidosis can progress to bacteremia and severe septicemia as in this patient. Acute kidney injury is also one of the complications seen in severe melioidosis [13]. Delay in considering melioidosis as a differential diagnosis and delay in initiating proper treatment for melioidosis can contribute to severe melioidosis.

Definitive diagnosis requires identification of *Burkholderia pseudomallei* in clinical specimens ideally by using Ashdown broth [14, 15]. Bacterial culture has 100% specificity in diagnosing melioidosis; however, sensitivity has been estimated as low as 60% in one Bayesian model [16]. The manual identification kits and automated microbial identification system generally do not recognize the organism as *Burkholderia pseudomallei* [5]. Therefore, it is essential to have high degree of suspicion of *Burkholderia pseudomallei* to look for Gram stain appearance, antibiotic susceptibility pattern, and colony morphology [5]. It is also difficult to isolate and identify *Burkholderia pseudomallei* from a nonsterile site due to scanty growth which is masked by the contamination with commensal flora. [4]. Ashdown's selective agar or an alternative selective agar is needed in addition to MacConkey and blood agar for primary isolation of *Burkholderia pseudomallei* from a nonsterile site [4]. Serological investigations play a role when cultures are negative [5].

*Burkholderia pseudomallei* shows resistance to many antibiotics [14]. It is sensitive to tetracycline, trimethoprim-sulfamethoxazole, chlorampenicol, 3^rd^ generation cephalosporin (ceftazidime), amoxicillin-clavulanic acid, ureidopenicillin, cefoperozone/sulbactam, azithromycin, and carbapenem [14]. This patient's isolate was resistant to ceftazidime but sensitive to meropenem and trimethoprim-sulfamethoxazole.

Current treatment guideline of melioidosis consists of two phases. They are two weeks of intensive therapy (which can be extended for 4 weeks if clinically required) with either intravenous ceftazidime or carbapenem followed by oral eradication therapy with trimethoprim-sulfamethoxazole for 3–6 months [15]. Rapid treatment of septicemia, eradication of the disease, and prevention of relapses are aims of this treatment regime [5]. Meropenem has some evidence in showing better outcome in severe melioidosis with septic shock [15].

Melioidosis causes significant mortality, morbidity, recurrences, and reinfection [5]. Therefore, early diagnosis and early initiation of correct antibiotic is necessary when managing a patient with melioidosis.

## 4. Conclusions

Melioidosis has a wide range of clinical presentations; therefore, it is necessary that clinicians have high degree of suspicion of melioidosis when a septic patient comes from a relevant geographical area with risk factors. It is important to have early suspicion and definitive diagnosis of melioidosis to start a correct treatment timely. This may reduce the morbidity and mortality in severe melioidosis.

## Figures and Tables

**Table 1:** Summary of investigation findings on day 01 and during ICU stay.

Investigation	Day 1	During ICU stay
1. WBC	14,000 cells/mm^3^ (88% of neutrophils)	23,000 cell/mm^3^ (neutrophil leukocytosis)
2. Haemoglobin	12.4 g/dl	11.8 g/dl
3. Platelet	204,000/mm^3^	112 × 103/mm^3^
4. CRP	173 mg/L	420 mg/L
5. ESR	18 mm/hour	20 mm/hour
6. ALT	21 U/L	251 U/L
7. AST	38 U/L	513 U/L
8. ALP)	81 U/L	171 U/L
9. Creatinine	92 micromoles/L	356 micromoles/l risen to 750 micromoles/L
10. Blood urea	18 mg/dL	81 mg/dl risen to 103 mg/dL
11. Serum sodium	138 mmol/L	140 mmol/L
12. Serum potassium	4.2 mmol/L	6.5 mmol/L
13. Creatinine phosphokinase		94 U/L
14. Total bilirubin		1.1 mg/dL
15. Prothrombin time	12 sec	38 sec
16. Activated partial thromboplastin time	38 sec	48 sec
17. International Normalized Ratio (INR)	1.3	2.7
18. Serum lactate		8 mmol/L

## Data Availability

The clinical data are available in the clinical records of the patient which are stored in the record room of Sri Jayewardenepura General Hospital, Nugegoda, Colombo, Sri Lanka.
